# Effect of Financial Stress and Positive Financial Behaviors on Cost-Related Nonadherence to Health Regimens Among Adults in a Community-Based Setting

**DOI:** 10.5888/pcd13.160005

**Published:** 2016-04-07

**Authors:** Minal R. Patel, Daniel J. Kruger, Suzanne Cupal, Marc A. Zimmerman

**Affiliations:** Author Affiliations: Daniel J. Kruger, Marc A. Zimmerman, Department of Health Behavior and Health Education, University of Michigan School of Public Health, Ann Arbor, Michigan; Suzanne Cupal, Genesee County Health Department, Flint, Michigan.

## Abstract

**Introduction:**

Little is known about the role of positive financial behaviors (behaviors that allow maintenance of financial stability with financial resources) in mitigating cost-related nonadherence (CRN) to health regimens. This study examined the relationships between positive financial behaviors, financial stress, and CRN.

**Methods:**

Data came from the 2011 Speak to Your Health! Community Survey (n = 1,234). Descriptive statistics were computed to examine financial stress and CRN, by chronic condition and health insurance status. We used multivariate logistic regression models to examine the relationship between positive financial behaviors and financial stress and their interaction on a composite score of CRN, controlling for health insurance status, educational level, age, marital status, number of chronic conditions, and employment status.

**Results:**

Thirty percent of the sample engaged in CRN. Participants reported moderate financial stress (mean, 13.85; standard deviation [SD] = 6.97), and moderate positive financial behavior (mean, 8.84; SD = 3.24). Participants with employer-sponsored insurance, Medicaid, Medicare, the Genesee Health Plan, high blood pressure, asthma, and diabetes had the highest proportion of CRN. The relationship between financial stress and CRN was not significantly different between those who reported lower versus higher levels of positive financial behavior (*P* = .32). Greater financial stress was associated with a greater likelihood of CRN (odds ratio [OR] = 2.49; 95% confidence interval [CI], 2.08–2.99). Higher level of positive financial behavior was associated with a lower likelihood of CRN (OR = 0.80; 95% CI, 0.67–0.94).

**Conclusion:**

Financial literacy as a means of promoting positive financial behavior may help reduce CRN. An intervention strategy focused on improving financial literacy may be relevant for high-risk groups who report high levels of financial stress.

## Introduction

One of 4 Americans reports financial difficulty in paying medical bills (1); this difficulty has significant public health implications, especially for the 50% of the population that is managing chronic illness. Seven systematic reviews concluded that several factors influence adherence to treatment, but cost to the patient is one that demonstrates a consistent negative effect (2). Nearly 18% of chronically ill Americans report underusing medications and delaying or not fulfilling therapeutic recommendations because of cost (3), which is referred to as cost-related nonadherence (CRN) (3) and varies by therapeutic class across chronic therapies (3,4). Fifty-six percent of American adults with common chronic diseases self-report nonfulfillment of medication as a result of financial hardship (5).

Health insurance coverage is a strong predictor of financial burden (6,7). Nearly half of Americans have literacy challenges with health insurance and pay more for health care out of pocket because of these challenges, despite improvements as a result of the Affordable Care Act (ACA) (8).

Although health literacy and health insurance literacy are commonly discussed as integral for individuals to have the capacity to obtain, process, and understand basic health information or services and health insurance, financial literacy in the context of health has received little attention. Financial literacy is a set of skills and knowledge that allows individuals to make informed decisions with their financial resources (9), and it is associated with more frequent engagement in health-promoting behaviors (10–12). Studies show that social determinants of health that contribute to financial burden correlate with CRN (13,14). Therefore, financial burden may be experienced in the context of a growing concern for financial insecurity and may not be exclusively health-related. Given the role that cost to the patient plays in adherence to therapeutic regimens, improving financial literacy to influence positive financial behaviors (behaviors that allow individuals to maintain financial stability with their financial resources) may have implications for CRN, and may be a necessary adjunct to policy reforms.

Few interventions have aimed to mitigate CRN beyond reducing out-of-pocket costs, which have shown modest improvements in health status (15). Whether positive financial behavior is protective of CRN has not been explored and may have implications for behavioral interventions to promote financial literacy, especially among people who have chronic illnesses.

We examined financial stress and CRN by type of chronic condition and health insurance status in a community-dwelling sample in Michigan. We also examined the relationship between financial stress, positive financial behaviors, and CRN, testing the hypothesis that the relationship between financial stress and CRN is different between people who report lower numbers versus higher numbers of positive financial behaviors.

## Methods

### Study sample

Data came from the 2011 wave of the Speak to Your Health! Community Survey administered to residents of Genesee County, Michigan (16). Genesee County is among the most economically disadvantaged counties in Michigan (17). We collected data from October 2011 through March 2012. The survey’s purpose was to monitor and understand local health concerns to inform efforts to improve the health of Genesee County communities and was developed by the team through a community-based participatory research process (18). All study procedures were approved by the University of Michigan Institutional Review Board.

Random samples of households were drawn from 129 Genesee County residential census tracts in an attempt to include at least 20 residents from each tract in Flint, Michigan, and 10 residents in suburban and rural census tracts. Inclusion criteria included being at least aged 18 and a resident of Genesee County. More details on the Speak to Your Health! Survey are available elsewhere (16,18,19). From 9,944 telephone listings, 1,234 respondents provided complete data. Inability to contact participants because of telephone issues totaled 10.9% (n = 1,088) of all attempted records. The response rate was 25% (1,234 of 4,936). Telephone numbers that were not in service or were never answered after 10 attempts (not including answering machines) were not included in response rate calculations. The complete and analytic samples were similar in age, years of education, sex, and body mass index (data not shown). All participants provided informed consent before data collection. Professional survey staff conducted a 25-minute computer-aided telephone interview (CATI) with randomly selected respondents.

### Measures

The outcome of interest in this study was CRN, a composite binary measure (yes/no) of a positive response to at least 1 of 2 items. The first item was not seeing a doctor because of cost, which was measured by asking participants, “Was there a time during the last 12 months when you needed to see a doctor, nurse, or other health professional but could not because of cost?” The second item was not filling a prescription because of cost, which was measured by asking participants, “Was there a time during the last 12 months when you needed to fill a prescription but could not because of cost?” (20).

Participants were asked a series of 9 questions to determine level of financial stress and use of positive financial behaviors. Items included whether participants set aside money from each pay period for savings, have trouble sleeping because of financial problems, are concerned because they cannot afford health insurance, and only spend what they can afford. All items were assessed on a 5-point Likert scale (1 being strongly disagree to 5 being strongly agree) (21,22).

To determine whether participants had any of 8 prevalent chronic conditions that require routine medical management (high blood pressure, heart disease, cancer, diabetes, asthma, sarcoidosis, sickle cell anemia, and lupus), participants were asked if they had ever been diagnosed by a doctor as having any of the conditions (yes/no). We developed this list on the basis of conditions with the highest overall health burdens and the interests of community-based organization partners. To assess the type of health insurance participants had, they were asked if they had a health insurance policy through the following mechanisms: employer-sponsored, nonemployer/private, Medicaid, Medicare, Genesee Health Plan, other, and no insurance coverage (yes/no) (20).

Analyses were conducted using SAS version 9.4 (SAS Institute, Inc). We used a principal-axis factor analysis with a varimax rotation for the 9 survey items related to finances to produce 2 orthogonal factors of interest (eg, financial stress and positive financial behaviors). Question items with factor loadings greater than 0.50 were included in a factor. Internal consistency was evaluated with the Cronbach α statistic for each of the final 2 orthogonal factors produced. Health-related financial stress and positive financial behavior items were both summed to create a score. The range of scores for financial stress was 1 to 30, with 1 indicating low stress and 30 indicating high stress. The range of scores for positive financial behaviors was 1 to 15, with 1 indicating low level of positive financial behaviors and 15 indicating high level of positive financial behaviors.

Descriptive statistics were computed for all demographic characteristics to examine the frequency of CRN and financial stress by chronic condition and type of health insurance. Multiple variable logistic regression analyses were used to examine the independent association between financial stress and positive financial behaviors (main independent variables) and CRN (dependent variable). Both of the variables for financial stress and positive financial behaviors were centered at the mean to standardize values and were included in the model as an interaction term to test the hypothesis that the relationship between financial stress and CRN is different between those who report lower levels of positive financial behaviors and those who report higher levels of positive financial behaviors. All models were adjusted for health insurance status, educational level, age, marital status, number of chronic conditions, and employment status.

## Results

The mean age of the sample (N = 1,234) was 53.5 years (standard deviation [SD] = 15.2 y), 73% were female, 47% were married, and 70% reported their race/ethnicity as white (Table 1). Ninety percent of the sample was employed, and 30% had an educational attainment of college or more. Nearly half of participants reported having employer-sponsored health insurance coverage and high blood pressure. Twenty percent of participants did not see a doctor, and 24% did not fill a prescription because of cost, resulting in a 30% prevalence of CRN.

**Table 1:** Demographic and Clinical Characteristics of the Sample (N = 1,234), Speak to Your Health! Community Survey, Michigan, 2011^a^

Factor	Value
**Mean age (standard deviation), y**	53.5 (15.2)
**Married**	554 (47)
**Employed**	1,105 (90)
**Educational attainment**	
≤High school	309 (26)
Some college/technical/associates	526 (44)
College or more	353 (30)
**Health insurance status^b^**	
Employer-sponsored	602 (49)
Private policy	84 (7)
Medicaid	185 (15)
Medicare	352 (29)
Genesee Health Plan	126 (10)
Other	113 (9)
No insurance coverage	71 (6)
**Female**	876 (73)
**Race/ethnicity**	
White	832 (70)
Black or African American	280 (24)
Other	75 (7)
**Body mass index, mean (standard deviation) kg/m^2^**	29.8 (7.7)
**Cost-related nonadherence^c^**	374 (30)
**Chronic condition^b^**	
High blood pressure	525 (45)
Heart disease	165 (15)
Cancer	139 (12)
Diabetes	227 (20)
Type 1	19 (9)
Type 2	183 (91)
Asthma	195 (18)
Sarcoidosis	16 (1)
Sickle cell anemia	14 (1)
Lupus	16 (1)
**No. of chronic conditions**	
0	445 (37)
1	380 (32)
≥2	363 (31)

a Values expressed as no. (%), unless otherwise indicated. Not all categories add to total, because some survey respondents did not answer all questions (percentages based on number of respondents who answered the question).

b Respondents could choose more than one answer.

c Twenty percent of participants did not see a doctor and 24% did not fill a prescription because of cost, resulting in a 30% prevalence of cost-related nonadherence in this sample.

Inter-item correlations among the 9 items that determined levels of financial stress and positive financial behaviors ranged from 0.07 to 0.83 (Table 2). Financial stress had strong reliability (6 items; Cronbach α = 0.89), and positive financial behaviors had adequate reliability (3 items; Cronbach α = 0.63). Participants reported a mean financial stress score of 13.85 (SD = 6.97) indicating moderate financial stress, and a mean score of 8.84 (SD = 3.24) for positive financial behaviors, indicating moderate positive financial behaviors. Financial stress and positive financial behaviors were not significantly correlated (*r *= 0.31).

**Table 2:** Characteristics of Financial Items From Exploratory Factor Analysis, Speak to Your Health! Community Survey, Michigan, 2011

Survey Item	No. Replied Yes (%)	Loadings	
		Financial Stress	Positive Financial Behaviors
Each pay period, I set aside at least 10% of my pay for savings.	327 (33)	0.13	0.77
I only spend what I can afford to spend.	927 (78)	0.28	0.67
I pay off my entire credit card bill each month.	436 (49)	0.21	0.75
I have trouble sleeping because of my financial problems.	186 (19)	0.74	0.08
I am concerned because I cannot afford adequate health insurance.	243 (26)	0.75	0.14
I often worry about my financial situation.	498 (45)	0.77	0.07
My financial situation is much worse this year than it was in the previous year.	510 (47)	0.79	0.27
I do not know how I will be able to support myself in the next 12 months.	194 (19)	0.83	0.07
How difficult is it for you to live on your total household income right now? (% high difficulty)	232 (19)	0.76	0.28
**Cronbach α**	NA	0.89	0.63

Abbreviation: NA, not applicable.

**Figure 1:**
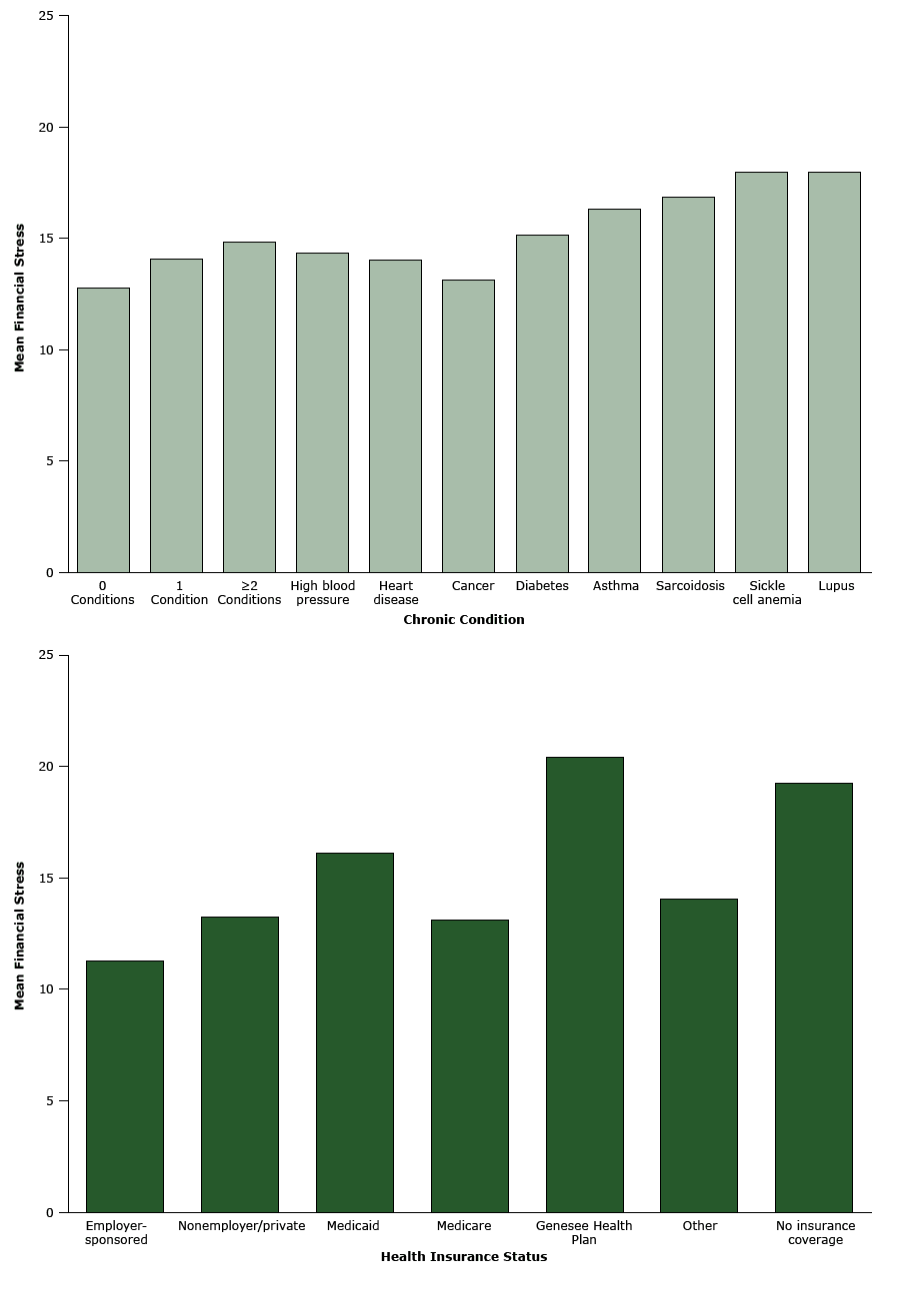
Mean level of financial stress, by chronic condition and health insurance status. Mean level of financial stress is a composite score based on 6 items; scores ranged from 1 to 30, with 1 indicating low levels of stress and 30 indicating levels of high stress. Speak to Your Health! Community Survey, 2011. **Table:** 

Figure 1 shows mean financial stress by number and type of chronic conditions and health insurance status. Moderate financial stress was evident among participants reporting diabetes, asthma, sarcoidosis, sickle cell anemia, lupus, 2 or more chronic conditions, coverage by the Genesee Health Plan, and no insurance coverage. 

Variable	Mean Financial Stress	
	No. of Survey Respondents	Value (Standard Deviation)
**Chronic condition**		
0 Conditions	443	12.79 (6.64)
1 Condition	373	14.09 (7.1)
≥2 Conditions	355	14.87 (7.08)
High blood pressure	525	14.38 (7.12)
Heart disease	165	14.06 (7.35)
Cancer	136	13.16 (6.57)
Diabetes	227	15.16 (7.27)
Asthma	193	16.34 (7.14)
Sarcoidosis	16	16.87 (6.38)
Sickle cell anemia	14	18.00 (8.06)
Lupus	16	18.00 (6.96)
**Health insurance status**		
Employer-sponsored	595	11.26 (5.85)
Nonemployer/private	83	13.25 (6.67)
Medicaid	183	16.13 (6.95)
Medicare	344	13.13 (6.47)
Genesee Health Plan	124	20.41 (6.56)
Other	109	14.05 (6.87)
No insurance coverage	71	19.23 (6.11)

**Figure 2:**
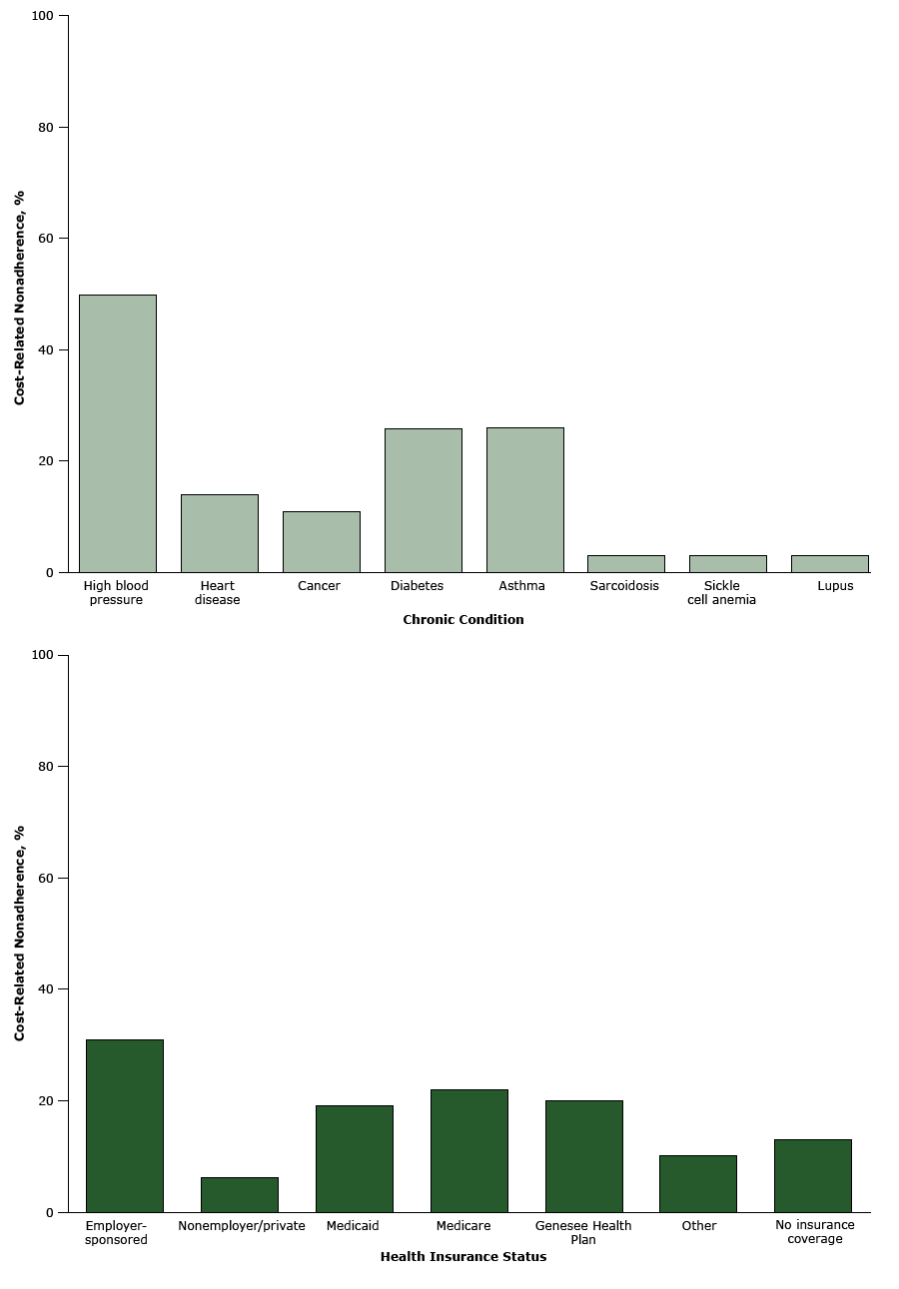
Cost-related nonadherence behaviors, by chronic condition and health insurance status. Cost-related nonadherence is a composite binary measure of a positive response to 1 of 2 cost-cutting behaviors with the treatment regimen. Speak to Your Health! Community Survey, Michigan, 2011. **Table:** 

Figure 2 shows CRN by chronic condition and health insurance. The highest proportion of individuals who reported CRN were those who had employer-sponsored health insurance, Medicaid, Medicare, the Genesee Health Plan, high blood pressure, asthma, or diabetes.

Variable	Cost-Related Nonadherence, %
**Chronic condition**	
High blood pressure	50
Heart disease	14
Cancer	11
Diabetes	26
Asthma	26
Sarcoidosis	3
Sickle cell anemia	3
Lupus	3
**Health insurance status**	
Employer-sponsored	31
Nonemployer/private	6
Medicaid	19
Medicare	22
Genesee Health Plan	20
Other	10
No insurance coverage	13

Multiple variable logistic regression analyses indicated that the interaction of financial stress and positive financial behaviors was not significant (*P* = .32), so it was dropped from the final model. Greater financial stress was associated with a greater likelihood of CRN behavior (odds ratio [OR] = 2.49; 95% confidence interval [CI], 2.08–2.99). Higher levels of positive financial behavior were associated with a lower likelihood of CRN (OR = 0.80; 95% CI, 0.67–0.94) (Table 3).

**Table 3:** Association Between Positive Financial Behaviors and Financial Stress on a Composite Score of Cost-Related Nonadherence, Speak to Your Health! Community Survey, Michigan, 2011^a^

Factor	Cost-Related Nonadherence, Odds Ratio (95% Confidence Interval) (N = 1,114)	*P* Value
**Positive financial behaviors**	0.80 (0.67–0.94)	.008
**Financial stress**	2.49 (2.08–2.99)	.001
**No health insurance**	0.47 (0.26–0.85)	.01
**Education**		
Some college/technical/associates	1.74 (1.12–2.70)	.01
College or more	1.77 (1.21–2.59)	.003
**Age**	0.96 (0.95–0.97)	.001
**Not married**	0.95 (0.69–1.30)	.76
**No. of chronic conditions**	1.35 (1.16–1.56)	.001
**Unemployed**	0.66 (0.41–1.05)	.08

a Models adjusted for the provision of health insurance, education, age, marital status, number of chronic conditions, and employment.

## Discussion

Many factors motivate adherence to a therapeutic health regimen, and this analysis focused on the role of cost as a factor. To our knowledge, this is the first study to examine the association between positive financial behaviors and CRN. Positive financial behaviors were moderate in this study of community-dwelling adults in an economically disadvantaged county in Michigan. The relationship between financial stress and CRN was not significantly different between those who reported lower versus higher levels of positive financial behavior in our sample; therefore, our hypothesis was not supported. This relationship requires further investigation in other samples. We found, however, that regardless of health insurance, higher levels of positive financial behaviors were associated with a lower likelihood of CRN, suggesting that positive financial behaviors may influence CRN. Our findings show an association between behavioral practices related to finances that influence CRN, whereas other work has shown that higher levels of health and financial literacy can promote health through improved health care decision making and behavior (11,12).

Researchers that found effects of financial literacy on health and health care decision making investigated financial literacy through measures that assess comprehension of financial concepts and numeracy skills based on mathematical computation (10–12). In our study, we examined a set of behaviors for effective financial management in the interest of long-term financial well-being that include saving, spending within means, and paying off debt. Our findings expand our understanding of the influences of financial literacy on health by considering functional financial literacy and a set of skills and behaviors related to financial management that may also be protective against the negative consequences of health-related financial burdens on health behaviors. This is different from health literacy or health insurance literacy, which may encompass knowledge, behaviors, and self-efficacy around general or specific areas of health or health insurance.

Improving financial literacy as an intervention strategy as a means to promote positive financial behavior to prevent CRN may be especially relevant for certain high-risk groups. Although we found an association between financial stress and CRN regardless of health insurance status and number of chronic conditions, our descriptive findings and other work (3) suggest that attention to financial literacy may be relevant for certain conditions in which levels of both financial stress and CRN are high: high blood pressure, asthma, and diabetes. The adequate prevention of adverse events for high blood pressure, asthma, and diabetes through a medication regimen can have implications for the development of severe comorbidity and poor long-term health, as well as increased financial burden. We are not aware of any evidence-based self-management programs for any of these conditions that incorporate skills training in financial literacy to promote positive financial behaviors or have an emphasis on reducing cost-related barriers to prevent CRN. Although the chronic conditions included in this study may differ by typical cost of treatment, this variance my affect individuals in terms of perceived financial stress based on the type of health insurance they have and what services are covered in their plan.

Although individuals with employer-sponsored health insurance in our sample reported the lowest levels of financial stress, rates of CRN were similar for those with local insurance options for low-income families (Genesee Health Plan) and those with no insurance, both of whom reported high levels of financial stress. Between 2007 and 2010, per-person out-of-pocket spending grew most rapidly for people primarily covered by employer-sponsored insurance because of a greater shift to high-deductible plan offerings by employers (23), which may encourage CRN. To date, no significant changes as a result of the ACA have been noted on consumer behavior among those with employer-sponsored insurance (24), suggesting that strategies to improve financial literacy may also be relevant for this group to prevent CRN.

Our study has several limitations. This was a secondary analysis of available survey data. Lack of household income data limited our interpretation of results to perceived financial burden. Median household income in Genesee County is $42,089, which is lower than state and national averages, suggesting economic burden in the region (25). Items used to generate constructs of financial stress and positive financial behaviors were based on available data. The items used may not capture all aspects of either construct, which may increase their content validity. Nevertheless, the measures have adequate reliability and capture key aspects of the construct, and the results were in the hypothesized direction. Adherence is also influenced by factors beyond cost; however, we did not have measures in our survey to examine other factors that influence adherence. We also did not have access to data to verify the CRN reported in this study against pharmacy claims or medical records, which would strengthen confidence in self-reported data. However, the rates of CRN reported in this study align with national averages and those reported elsewhere (3,5,7), so the self-reported nature of the data was not a major concern for our confidence in the results. Another limitation was that participants were from one economically disadvantaged county in Michigan. Furthermore, women and older adults were overrepresented in the sample; as a result, findings may not be generalizable nationally or to Michigan as a whole, but they may be generalizable to other economically disadvantaged communities. Future work that considers replicating these analyses with nationally representative data would be useful. Finally, the data for the study were collected when the ACA was first passed. Rates of the provision and type of health insurance may have shifted since the enactment of ACA reforms in the region. However, the Genesee Health Plan was available before the ACA to provide basic health insurance for uninsured, low-income adults (26); therefore, the differences in the rate of the insured versus uninsured before and after the ACA may not differ. Although we did not have information for types of health insurance, our results reflect current trends of financial stress based on cost sharing that are consistent with the data reported here.

Despite these limitations, this study has implications for behavioral interventions. Policy interventions may intend to reduce financial stress from medical bills, but they do not provide the requisite skills for individuals and families to understand and therefore effectively manage the financial elements of their health care. Literacy and numeracy may have significant influence on how people choose cost-effective health plans and manage out-of-pocket expenses. These skills may be especially salient for those who manage chronic diseases, which require lifetime management with a therapeutic regimen and routine interface with the health care system. Building health literacy around the importance of adherence is imperative. Because financial literacy may protect against CRN, behavioral interventions for chronic diseases that teach and facilitate self-management skills may also consider skills training in health-related financial literacy. This may be a vital direction for intervention design, because financial stress was predictive of CRN despite health insurance status and number of chronic conditions. Improving financial literacy to promote positive financial behavior may be relevant for certain high-risk groups who report high levels of financial stress, such as those managing high blood pressure, asthma, or diabetes, or who have health insurance plans with high or variable cost sharing.
